# Incidence and determinants of stillbirth among women who gave birth in Jimma University specialized hospital, Ethiopia

**DOI:** 10.11604/pamj.2017.28.299.1269

**Published:** 2017-12-08

**Authors:** Dejene Tilahun, Tsion Assefa

**Affiliations:** 1 Jimma University, Institute of Health, Faculty of Public Health, Department of Health Education and Behavioral Sciences, Jimma, Ethiopia

**Keywords:** Stillbirth, incidence, determinants, risk factors, protective factors, Ethiopia

## Abstract

**Introduction:**

Worldwide approximately 2.7 million are stillborn, more than 98% of these occur in developing countries. To address the problem, incidence and determinants of stillbirth must be understood. Therefore the aim of this study was to assess incidence and determinants of stillbirth among women who gave birth in Jimma University specialized hospital.

**Methods:**

A cross-sectional study design among 413 mothers who gave birth in Jimma specialized hospital was employed. Study subjects were selected by systematic sampling technique from the list of women who gave birth in hospital in one month study period. Data were collected by using pretested and structured questionnaire. Data were edited, cleaned, coded, entered and analyzed using SPSS-20 statistical software. Univarate and bivariate (logistic regressions) analysis was employed.

**Results:**

The incidence rate of stillbirth in the Hospital during a month period was 8% or 80 per 1000 total births. The predictors that showed an independent close association with stillbirth were absence of complication (OR = 0.1, 95% CI (0.04-0.2)), referral from other health facility (OR = 0.3, 95% CI (0.1-0.7)), having antenatal care (OR = 0.3, 95% CI (0.1-0.7)) and normal vaginal delivery (OR = 0.2, 95% CI ( 0.1-0.8)).

**Conclusion:**

The incidence rate of stillbirths in our setting is high and the identified determinants were related to both ante-partum and intra-partum-period. Therefore, effort should be made to improve antenatal, obstetric services and delivery services in terms awareness, access, timing and referral system to emergency care and specialized service to reduce the number of stillbirths.

## Introduction

Despite increasing attention and investment for maternal, neonatal, and child health, stillbirths remain invisible i.e. not counted in the millennium development goals, nor tracked by the united nation, nor in the global burden of disease metrics. At least 2.7 million stillbirths were estimated worldwide and more than 98% of these occur in low-income and middle-income countries (LAMIC) [1, 2]. Depending on access to and quality of obstetric care, as well as prevalence of antenatal risk factors, the proportion of stillbirths may vary from country to country [2]. The average stillbirth rate in low and middle income countries (LAMIC) has been reported to be 26 per 1000 live births, about five times higher than in high income countries (HIC) (5 per 1000) [3]. Countries in Africa report the highest incidence rates per 1000 live births [2-4]. Despite an important indicator stillbirths are invisible in global policy and programme priorities, they are usually not captured in local data collecting systems [2-4]. To address the problem of still birth in LAMIC, incidence and determinants of stillbirth must be understood. This is because incidence of stillbirth is an important indicator of the quality of antenatal and obstetric care [2, 4, 5]. And most risk factors of stillbirth that occurs during ante partum and intra partum period in are preventable [3]. Several studies in LAMIC [6-18] suggests different determinants for stillbirths. These includes lack of adequate access to obstetric care, inadequate care, maternal infections and complications like antepartum bleeding and pregnancy-induced hypertensive disease, poor care during labour and delivery, fetal growth restriction and congenital abnormalities AND socio-demographic characteristics (age, parity, religion, residence and healthcare) are the most important risk factors, while some causal pathways remain unknown. Hence stillbirth rates can be considered a proxy for access to and quality of reproductive health and ANC services and care during labour and delivery [5].

Although studies have identified a number of determinants of still birth, in both the HIC and LAMIC there is very little published literature that focuses on the determinants of still birth in Ethiopia, particularly on determinants of still birth among women that gave birth in hospital. Moreover, in most of the studies of LAMIC, association between stillbirth and socio-demographic characteristics, obstetric and reproductive has been examined largely based on data collected only retrospectively [5-11, 14-22]. Studies in Zimbabwe, Nigeria, Ghana and Gambia [7, 9, 11] indicate that women delivering stillbirths were less likely to receive prenatal care (ANC) than those not delivering stillbirths. The same finding in Nepal, Zimbabwe and Nigeria showed that those who gave birth with caesarian section have less likely to have still birth compared to those who have not [7, 9]. And the risk of still birth was more common in vaginal delivery assisted with instrument as compared to normal vaginal delivery [7, 11, 12, 17]. Studies in Gambia, Zimbabwe, Tanzania and China [5, 9, 18] indicate an increase likelihood of still birth in those women who come from rural area and peripheral area. Still birth was less common in women who have severe complication than those who have no complication [7, 9, 11, 15].

Therefore data on the frequency and determinants of still birth are important for planning maternal and child health care services in developing countries and can enable policymakers and program planners to design legislation and services of the reproductive health program which will decrease frequency as well as the risk factors of women who are most likely to experience this problem. In this preliminary study, we assessed the contribution of demographic and reproductive/obstetric risk factors to the rate of stillbirth in Jimma University specialized and teaching Hospital, Ethiopia.

## Methods

Facility based cross sectional study was conducted at Jimma University specialized Hospital maternity unit in the department of obstetrics and gynecology in south-west Ethiopia. The hospital is the only teaching and referral hospital for south western part of Ethiopia and gives different specialized clinical services including maternal and child service for about 15 million population including referral cases from different region including the South Sudanese refuges. The maternity unit in the Department of Obstetrics and Gynecology at the hospital has between 4 and 8 specialist physicians. There are between four and eight nurse and midwives nurse for each shift. The department also has specialty training in obstetrics and gynecology residents which took four year to finish the specialty training that means in the departments there are resident specialty from 1^st^ year to 4^th^ year. Jimma University specialized Hospital is a 500 bedded hospital with 45 maternity beds, 5 delivery tables. During admission, detail history regarding age, parity, obstetric history and other reproductive health status are taken. Regular checks up are done by obstetrician, resident obstetricians and medical interns. Most deliveries are conducted by resident doctors (obstetricians) with the help of midwife/nurses on duty. The sample size was determined using single population proportion formula assuming; 95% level of confidence, 50% proportion of antenatal care (ANC) and non-response of 10%. This made the final sample size 422 women. A systematic sampling technique was used to identify study participants. Participant mothers were identified using delivery registration record book. Every one mother who gave birth or delivered was interviewed, until the required sample size was attained. The data were collected using structured questionnaires and check lists which were adapted from similar survey used by similar studies [9, 11]. The questioners contain the following parts: Socio demographic factors (respondents' age, marital status, religion, ethnicity, education status, income, the number of children a woman has, occupation and other information). Obstetric and reproductive factors (ANC follow up, number of pregnancy, parity, means of transport to hospital, cause of complication mother experienced). Infant and delivery related issues (history of still birth, type of delivery, delivery outcome of the mother, delivery outcome of the fetus, type of skilled personnel).

The data were collected using pre-tested structured questionnaires which were adapted from similar survey used by similar studies [9, 11]. The questionnaires were prepared in English and translated in to Amharic and retranslated back to English to check its consistency. The interview was conducted after delivery and before the mother left delivery unit. All mothers were informed about the purpose of the study, importance of their participation and consent was ensured. Based on their willingness to participate in the study, they were interviewed by the interviewer. After they have completed the interview, the questionnaires were returned to the supervisors. Data collectors were recruited from other nearby institutions who were working in delivery unit. Training for data collectors and supervisors were given for two days, to make them familiar with the study instrument, consent form, how to interview, where to interview, when to interview, and on data collection procedure in general, by the principal investigator. All filled questioners were checked daily for completeness, accuracy, clarity and consistency by the supervisors and investigator. Necessary correction and changes were made on time. The data was cleaned, checked for inconsistencies and missing values, coded and entered in to statistical package for social sciences (SPSS) for versions 20.0. The data was organized and presented by using tables and frequencies to see the overall distribution of the study subject with the variables under study. For bivaret analysis, crude odds ratio was computed to assess the presence and degree of association between different variables with 95% confidence interval. Alpha value of 0.05 was considered for statistical significance. Logistic regression analysis was used to identify the independent risk factors or predictors variables on still birth. Research ethical clearance and approval was obtained from ethical clearance committee of Public Health and medical science college, Jimma University after submission of the proposal. A written consent was obtained from Jimma specialized hospital. All the study participants were informed about the purpose of the study and their consent were obtained before interview. Written informed consent was obtained from every study subject before the interview by explaining the objective of the research. All the information collected from the study subjects was handled confidentially through omitting their personal identification, conducting the interview in private place and the data were used for the research purpose only.

## Results

The response rate of respondents who gave birth was 97.8%. The mean age of participants was 25.1 ± 4.9 years. Majority, 269 (65.1%) of the women were Oromo. More than half, 240 (58%) of respondents were Muslim. Most 302 (73.1%) and 390 (94.7%) women were literate and married respectively. Nearly half (52.1%) of participants were house wives (Table 1). When respondents obstetric and reproductive condition were asked and assessed, nearly half (50.8%) of women were primiparas (first pregnancy) and (49.2%) women were multiparas (previous pregnancy). The mean number of live births among participants was 2.2 children. One hundred (24.2%) study participants had previous history of stillbirth. From the total birth of the delivered infants 33(8%) were stillbirths, representing a stillbirth rate of 80 per 1000 births. Most, 353(85.5%) women were attended antenatal care at least once (Table 2). Most, 259 (62.7%) women gave birth with spontaneous vaginal delivery. More than half (55.9%) of the respondents were attended by the resident specialists of obstetricians and gynecologist. Three hundred thirty two (80.4%) of the delivery were normal and the rest 81(19.6%) women were delivered with complication. The most serious cause of maternal complications during delivery was bleeding (58.02%) and sepsis (38.3%). Two hundred fourthy eight (60%) deliveries were referred from other health facility (Table 3). The crude analysis indicate that stillbirths were significantly associated with the following factors: Having awareness on unforeseen problems during pregnancy/child birth, (COR = 0.4, 95% CI (0.2,0.9)), educational status of husband (COR = 3.3, 95% CI (1.6-6.9)), referral from a peripheral health facility (COR = 0.3,95% CI (0.1-0.6)), obstetric complication (COR = 0.1, 95% CI (0.04-0.2)), antenatal care (COR = 0.2, 95% CI (0.1-0.4)), normal vaginal delivery (COR= 0.2, 95% CI (0.1,0.5)) and assisted vaginal delivery (COR = 0.4 , 95% CI (0.2-0.9)) (Table 4). After adjusted for all the variables in a logistics regression analysis, the factors independently associated with stillbirth were: normal delivery outcome (delivery without complications) (AOR = 0.1, 95% CI (0.04-0.2)), referral from a health facility (AOR = 0.3, 95% CI ( 0.1-0.7)), normal vaginal delivery (AOR = 0.2, 95% CI (0.1-0.8)) and having ANC follow up (AOR = 0.3, 95% CI (0.1-0.7)) (Table 4).

**Table 1:** Socio-demographic characteristics of women who gave birth at JUSH, South West Ethiopia

Socio demographic characteristics	Frequency	Percent
**Age (n=413)**		
<20 years	39	9.4
20 to 34 years	348	84.3
35 to 45 years	26	6.3
**Religion**		
Christian	173	41.9
Muslim	240	58.1
**Ethnicity**		
Oromo	269	65.1
Amarha	69	16.7
Guragie	29	7.0
Dawero	27	6.6
yem	19	4.6
**Marital status**		
Single	23	5.3
Married	390	94.7
**Educational status**		
Illiterate	111	26.9
Elementary	82	19.8
Secondary and above	220	53.3
**Mother occupation**		
Farmer	99	24.0
House wife	215	52.1
Government employer	46	11.1
Merchant	53	12.8
**Husband education**		
Illiterate	83	20.1
Literate	330	79.9

**Table 2:** Obstetric related and fetal related condition of study participants, Jimma University Specialized Hospital, South West Ethiopia

Variables	Frequency	Percent
**First pregnancy**		
Yes	210	50.8
No	203	49.2
**Number of pregnancies**		
Gravida one	205	49.6
2 to 4	161	39.0
5 and above	47	11.4
**ANC attendance**		
Yes	353	85.5
No	60	14.5
**History of stillbirths**		
Yes	100	24.2
No	313	75.8
**Still birth during index pregnancy**		
Yes	33	8.0
No	380	92.0
**Unforeseen problems during pregnancy/child birth**		
Yes	276	66.8
No	137	33.2
**Referred from peripheral health facility**		
Yes	248	60.0
No	165	40.0

**Table 3:** Delivery related conditions of respondents, Jimma University Specialized Hospital, South West Ethiopia

Variables (n=413)	Number	Percent (%)
**Type of delivery**		
Vaginal normal	259	62.7
Vaginal assisted	26	6.3
Cesarean section	128	31.0
**Who had attended the delivery**		
Residence of obstetricians	221	53.5
Medical interns	123	29.8
Midwife/nurse	69	16.7
**Means of transport to hospital for delivery**		
On foot	39	9.4.0
Vehicle transport	374	90.6
**Delivery outcome of infant**		
Alive	380	92.0
Still birth	33	8.0
**Delivery outcome of mother**		
Normal	332	80.4
Complicated	81	19.6
Type of complication*		
Bleeding	47	58.0
Sepsis	31	38.3
High B/P	16	19.8
uterine rupture	9	11.0
**Referred from peripheral health facility**		
Yes	248	60.0
No	165	40.0

* Multiple response

**Table 4:** factors independently associated with occurrence of stillbirth among pregnant mother who delivered in Jimma University Specialized Hospital, South West Ethiopia

Variables	Stillbirth		COR	AOR
	Yes	No		
**Educational status of mother**				
Illiterate	12(36.4)	99(26.1)	1.6(0.8, 3.4)	0.4(0.1, 1.4)
Literate	21(63.6)	281(73.9)	1.00	1.00
**Husband education**				
Illiterate	14(42.4)	69(18.2)	3.3(1.6, 6.9)**	1.9(0.6, 5.5)
Literate	19(57.6)	311(81.8)	1.00	1.00
**Unforeseen problems during pregnancy/child birth**				
Yes	16(48.5)	260(68.4)	0.4(0.2,0.9)	1.0(0.3, 3.2)
No	17(51.5)	120(31.6)	1.00	1.00
**ANC follow up**				
Yes	17(51.5)	321(84.5)	0.2(0.1,0.4)**	0.3(0.1, 0.7)**
No	16(48.5)	59(15.5)	1.00	1.00
**Referred client**				
Yes	10(30.3)	238(62.6)	0.3(0.1,0.6)***	0.3(0.1, 0.8)*
No	23(69.7)	142(37.4)	1.00	1.00
**Type of delivery**				
Vaginal normal	13(39.4)	246(64.7)	0.2(0.1,0.5)**	(0.2, (0.1, 0.8)*
Vaginal assisted	6(18.2)	20(5.3)	0.4(0.2,0.9)*	1.0(0.4, 2.4)
Caesarian section	14(42.4)	114(30.0)	1.00	1.00
**Delivery outcome mother**				
Normal	10(30.3)	322(84.7)	0.1(0.04,0.2)***	0.1(0.04, 0.2)***
Complicated	23(69.7)	58(15.3)	1.00	1.00
**Awareness on BPCR**				
Yes	13(39.4)	199(52.4)	0.6(0.3, 1.2)	0.9(0.3, 3.0)
No	20(60.6)	181(47.6)	1.00	1.00

ANC: Anti natal care, BPCR: Birth preparedness and complication readiness

* P< 0.05

** P<0.01

*** P<0.001

## Discussion

The incidence rate of still birth in our study was (8%), that represented 80 per 1000 total birth, which was higher than deliveries at maternity hospital of Zimbabwe 56 per 1,000 births [9], Maiduguri teaching hospital, Nigeria 22/1000 [7], and north East Tanzania 41.1 per 1000 births [20]. This difference in our finding for higher still birth rates than other hospitals may be because of high flow of high risks, delayed and most complicated cases from different peripheral region of the country and health facility as it is the only referral hospital in the south west Ethiopia. Moreover, unavailability of transportation, lack of adequate knowledge on maternal health service and delay in seeking and receiving skilled care increases the risk of stillbirth [13]. However, our finding showed lower rate of still birth than other hospital deliveries in developing countries. For example, the rate of stillbirth in Gambia hospital was 156 per 1000 [11]. The difference may be because of the difference in ante partum and intra partum care in the study settings, the difference in early detection of severe diseases and complication (that made a lower still birth), the wide range of referral system and type of referred obstetric case could also cause a difference in the rate of still birth [1, 4].

Our finding on the determinants is comparable with the findings of many studies in LAMIC [3, 5-7, 9-11, 16,18, 22]. According to the current study women who received antenatal care had less likely to have stillbirth than those mothers who had not received ANC follow up. This study was in line with the studies conducted at Maternity Hospital of Zimbabwe, Nigeria, Ghana and Jamaica [7, 9, 10, 22]. This may be due to high opportunity to identify high risk pregnancies during the antenatal period and for early and proper action of any danger signs. And the finding suggests a need to focus and emphasize on prenatal care. The study also showed a decrement in still birth by 70% among those referred pregnant women from other health facility compared to those not referred. This finding is comparable with the study conducted in maternity hospital of Tanzania, Zimbabwe, Ghana, china and Jamaica [5, 9, 10, 18]. This implies a need to broaden and decentralize accessibility of specialized and emergency obstetric and maternity service for the target population. Compared to a caesarian section delivery, normal vaginal delivery were significantly associated with still birth, those mothers delivered with normal vaginal delivery was 80% less likely to have stillbirth than caesarian section delivery. This result was different from the other studies in developing countries [7, 9]. Those who had delivered with caesarian section were less likely to have still birth compared to those who had normal vaginally delivery. The difference may be because most referred mothers delivered with caesarean section were from rural peripheral facility and after getting complication (rupture of uterus, bleeding) and this may cause an increase stillbirth. Those women who had normal delivery outcome had 90% less risk to have stillbirth than those that had obstetric complications. This was consistent with the study conducted in other developing countries [7, 9, 11,15].

Our study has some limitations. The study was facility based and conducted on one hospital only, thus findings may not be representative of all hospital of Ethiopia and may not be generalized to women delivered at their home. Limitation of a cross-sectional study also limits our ability to draw causal or temporal associations. Moreover, social desirability bias i.e. since the study touches sensitive issues the possibility of underestimation cannot be excluded, even though the study was anonymous. On the other hand, the study has several strengths including being the first study among mothers who gave birth in the hospital. The high participation and consistency with other studies in some determinants of stillbirth can in turn increase reliability of the findings. The public health importance, potential policy implications and applicability of the findings to similar settings were also an important strength of the study.

## Conclusion

The incidence rate of stillbirths in our setting is high and the identified risk factors were related to both ante partum and intra partum periods. Therefore, effort should be made to improve antenatal, obstetric services and delivery services in terms of awareness, access, timing and referral system to emergency care and specialized service like cesarean delivery during labour and delivery to reduce the number of stillbirths. In general, the study comes up with a lot of relevant findings which has a policy implication to reduce the incidence rate of still birth through reduction of risk factors of still birth and enhancement of protective factors of still birth. Further follow up study need to be conducted in large population to determine incidence of stillbirth and determinants still birth in Ethiopia.

### What is known about this topic

- The prevalence of stillbirth has been reduced to a minimum unavoidable rate in developed countries; however it still remains very high in LAMIC; from total stillbirths in the globe, more than 98% of these occur in low-income and middle-income countries;
- The commonest determinants or causes of still birth are lack of prenatal care, prematurity, birth asphyxia, obstetric complications including ruptured uterus, maternal infections in pregnancy, antepartum hemorrhage, pregnancy-induced hypertension, fetal growth restriction and congenital abnormalities;
- Women who gave birth with caesarian section have less likely to have stillbirth compared to those who have not, and the risk of still birth was more common in vaginal delivery assisted with instrument as compared to normal vaginal delivery.

### What this study adds

- The incidence rate of stillbirth in the study setting was very high, 80 per 1000 total birth;
- The determinants of still birth in the study were maternal complication, antenatal care follow up, normal vaginal delivery and referral from other health facility;
- The likelihood of stillbirth was decreased by 90% among mothers who gave birth with no complication; the odds of stillbirth were decreased by 80% for normal vaginal delivery, and 70% for antenatal care follow up and referral from other health facility.

## Competing interests

The authors declare no competing interests.
